# The Translational Bridge between Inflammation and Hepatocarcinogenesis

**DOI:** 10.3390/cells11030533

**Published:** 2022-02-03

**Authors:** Sabine Gufler, Rita Seeboeck, Christoph Schatz, Johannes Haybaeck

**Affiliations:** 1 Institute of Pathology, Neuropathology and Molecular Pathology, Medical University of Innsbruck, 6020 Innsbruck, Austria; sabine.gufler@i-med.ac.at (S.G.); christoph.schatz@i-med.ac.at (C.S.); 2 Clinical Institute of Pathology, University Hospital St. Poelten, Karl Landsteiner University of Health Sciences, 3100 St. Poelten, Austria; rita.seeboeck@stpoelten.lknoe.at; 3 Department Life Sciences, IMC University of Applied Sciences Krems, 3500 Krems, Austria; 4 Diagnostic & Research Center for Molecular BioMedicine, Institute of Pathology, Medical University of Graz, 8010 Graz, Austria

**Keywords:** acute hepatitis, chronic hepatitis, hepatitis virus, alcoholic liver disease (ALD), non-alcoholic fatty liver disease (NAFLD), cirrhosis, steatosis, autoimmune hepatitis, hepatocellular carcinoma (HCC), mTOR signaling, eukaryotic translation initiation factors (eIFs)

## Abstract

Viral infections or persistent alcohol or drug abuse, together with intrinsic factors, lead to hepatitis, which often ends in the development of liver cirrhosis or hepatocellular carcinoma (HCC). With this review, we describe inflammatory liver diseases, such as acute liver failure, virus-induced hepatitis, alcoholic- and non-alcoholic steatohepatitis, and autoimmune hepatitis, and highlight their driving mechanisms. These include external factors such as alcohol misuse, viral infection and supernutrition, as well as intrinsic parameters such as genetic disposition and failure, in immune tolerance. Additionally, we describe what is known about the translational machinery within all these diseases. Distinct eukaryotic translation initiation factors (eIFs) with specific functional roles and aberrant expression in HCC are reported. Many alterations to the translational machinery are already triggered in the precancerous lesions described in this review, highlighting mTOR pathway proteins and eIFs to emphasize their putative clinical relevance. Here, we identified a lack of knowledge regarding the roles of single eIF proteins. A closer investigation will help to understand and treat HCC as well as the antecedent diseases.

## 1. Introduction

External influences such as viral infections or continuous alcohol or drug abuse, together with intrinsic factors such as gender, genetic disposition and the intestinal microbiome, induce inflammatory processes in the liver. By disrupting normal liver function, such as nutrient storage, lipid and glucose metabolism, and detoxification, chronic inflammation may lead to severe liver disorders and to the development of cirrhosis or hepatocellular carcinoma (HCC). HCC accounts for around 90% of all types of liver cancer [1], but its development can result from different causes. In addition, several studies have reported a crucial role of autophagy in liver injuries, which is negatively regulated by mTOR as a modulator of autophagy-associated proteins [2]. With this review, we describe inflammatory liver diseases and the different mechanisms of their development. Among the impacting parameters, we and others have demonstrated that eukaryotic translation initiation factors (eIFs) and the associated major regulator mTOR are particularly crucial molecular influencers. Thus, this review is also devoted to the role of eIF and mTOR pathway proteins in the clinical condition of inflammatory liver disease and HCC.

## 2. Acute Liver Failure

Acute liver failure (ALF) is loss of liver function occurring rapidly in a patient who very often has no preexisting liver disease. Triggers are frequently virus infections or drugs, such as acetaminophen [3]. The latter has recently demonstrated its known hepatotoxicity in many COVID-19-associated events of ALF after treatment [4]. Serious complications associated with ALF, which is always a medical emergency and also known as fulminant hepatic failure, include excessive bleeding and increasing pressure in the brain [3]. The most frequent cause of ALF is viral infection, with hepatitis A, B and E being equally responsible in developing countries and accounting for up to 70% of cases, whereas, for Europe, these numbers are declining and make up only about 15% of cases [5]. Infection with HAV and HBV can be safely prevented by vaccination. Though the virus itself would not be cytopathic, the immune response causes ALF in response to an infection. The current understanding is that cytokines, mainly interferon, eliminate the HBV genome within hepatocytes, before, at a later timepoint, T cells infiltrate the liver, essentially destroying hepatocytes [6]. Further Hepatitis Virus strains, such C or E, occur too seldomly to conclude specific mechanisms in association with ALF. This is also true for other virus classes, but there are reports linking ALF to infections with herpes simplex virus 1 and 2, human herpes virus type 6, cytomegalovirus, varicella virus, Epstein–Barr virus and parvovirus B19 and, less frequently, toga virus, paramyxovirus and parainfluenza virus [7].

Drug-induced ALF is caused by idiosyncratic drug reactions, including autoimmune responses. Acetaminophen is known to bear a larger risk for developing ALF and its mechanisms are described [8]. The trigger for ALF is the formation of toxic metabolites of cytochrome P450, which needs glutathione for clearance. If glutathione stores are depleted due to risk factors including age, gender, nutrition status, concomitant drugs, alcohol abuse or certain genetic predispositions preventing detoxification, liver injury may follow [9]. 

The prognosis of ALF is determined by the intrahepatic metabolic consequences, the load of toxic metabolites and the restorative capacity of the remaining hepatocytes [3]. Drug-induced liver injury management is exercised by a prompt cessation of the culprit drug. A therapy with corticosteroids is given in severe cases and in immune-mediated cases [8,9]. The underlying aim is to restore and to promote physiological liver regeneration. 

Although the link between ALF and cellular stress and, with that, eIF2α phosphorylation is suggested [10], no reports of an in-depth investigation were found. Similarly, the specific literature on mTOR signaling and ALF is rare and mainly focuses on allograft rejection or associated viral infections.

## 3. Viral Hepatitis

As a silent disease, chronic viral hepatitis often remains untreated. It is responsible for considerable global morbidity and mortality from secondary diseases such as liver cirrhosis and hepatocellular carcinoma [11]. Among the currently known hepatitis viruses A, B, C, D, and E, infections caused by the hepatitis B (HBV) and the hepatitis C virus (HCV) often lead to a chronic course [12]. Both pathogens are transmitted through blood or body fluids. As the main causes of cirrhosis, liver decompensation, HCC and liver transplantation, both viruses contribute to a considerable global burden on the health system, a decreased quality of life and an increased risk of mortality among infected individuals [13]. In recent years, some progress has been made in the prevention of chronic hepatitis. Prevention primarily succeeds by screening people at risk, vaccination, and avoiding hepatitis transmission via transfusions and injections [14].

In order to combat virus infections, a deep understanding of the complex interactions between virus and host must be acquired. Since viruses do not have their own metabolic and reproductive mechanisms, they are dependent on their host. Even for the first steps of infection, they use cellular transport mechanisms to enter the host cell or, in the case of DNA-or retroviruses, the cell nucleus. Viruses usually bring crucial enzymes with them for both successful integration into the host genome and the production of virus particles in the cytoplasm [15]. The eukaryotic translational machinery is the most prominent cellular process occupied by the virus to propagate its own genome. Therefore, this review will feature the proteins involved in the early steps of gene expression. 

### 3.1. Hepatitis A (HAV)

Hepatitis A virus (HAV) infection is one of the most common causes of acute hepatitis. It is mainly transmitted via the fecal–oral route through contaminated food or water or by close contact with infected people [16]. Unlike other hepatitis viruses, HAV does not cause chronic liver disease [17]. In areas that are highly exposed to HAV, most people acquire immunity to the virus in childhood [16].

**Figure 1:**
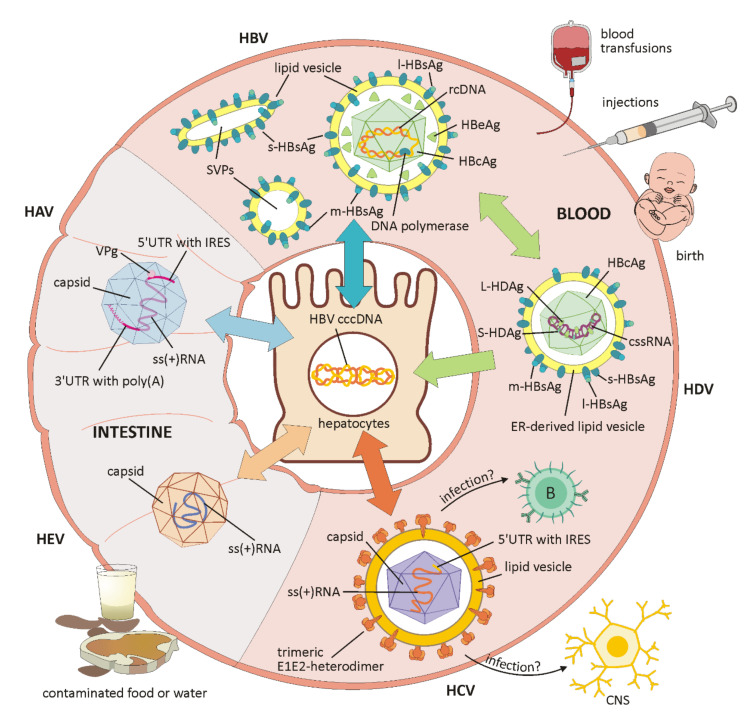
Structure and ways of transmission of the hepatitis viruses A, B, C, D and E. While HAV and HEV are enterically transmitted by contaminated water or food and mainly cause acute hepatitis, HBV, HDV and HCV are blood-borne infections, which are responsible for chronic disease development in many cases. (HAV) HAV has a single-stranded RNA genome encoding a polyprotein, which is translated 5′cap-independently under the control of an IRES. The 5′UTR is linked to VPg and the 3′UTR ends with a poly(A) tail. The virion exists as a non-enveloped form in the stool (as depicted here) and as eHAV in the blood of infected patients (not shown). (HBV) HBV exclusively infects liver cells and is packed in a lipoprotein envelope containing small, medium and large surface proteins. The core protein builds up a nucleocapsid containing partially double-stranded DNA (rcDNA) with an associated polymerase. In addition to viral particles, pre-core protein (HBeAg), and high amounts of non-infectious SVPs containing only envelope proteins are secreted. Inside the nucleus of infected hepatocytes, the HBV genome is converted to cccDNA, which remains stable in form of a mini chromosome or episome serving as a genomic template for viral mRNA synthesis. (HDV) HDV is known to infect hepatocytes and has a css(-)RNA that codes for only one viral protein in two forms, a small (S-HDAg) and a large (L-HDAg) viral antigen. By base-pairing, the genomic RNA has a rod-like structure and is associated with S-HDAg and L-HDAg, forming an RNP. In order to form virus particles, HBsAg envelope proteins of the HBV virus are embedded in an ER-derived lipid vesicle. (HCV) HCV is an enveloped virus with a single positive-stranded RNA encoding a polyprotein, which is subsequently cleaved into 3 structural and 7 functional proteins. It primarily infects hepatocytes, but HCV-RNA has been detected in peripheral blood cells, cerebrospinal fluid and in the brain of patients with chronic infection. Two envelope glycoproteins, E1 and E2, form trimeric heterodimers in the host-derived lipid envelope and are the main target of neutralizing antibodies during immune response. (HEV) HEV is mainly transmitted by contaminated food or water and replicates in the liver. In the stool and in the liver, it occurs as envelope-free virus with an ss(+)RNA encoding an enzymatic polyprotein and structural proteins. In the blood of infected patients, it can be detected as eHEV virion (not shown). Abbreviations (in alphabetical order): B = B lymphocyte; cccDNA = covalently closed circular DNA; CNS = central nervous system; css(-)RNA = circular single negative-stranded RNA; eHAV = quasi-enveloped hepatitis A virus; eHEV = quasi-enveloped hepatitis E virus; ER = endoplasmic reticulum; HBcAg = hepatitis B core antigen (capsid protein); HbeAg = hepatitis B e antigen (precore protein); IRES = internal ribosomal entry site; l-HbsAg = large hepatitis B surface antigen; L-HDAg = long hepatitis D antigen; m-HbsAg= medium hepatitis B surface antigen; rcDNA = relaxed circular DNA; RNP = ribonucleoprotein; s-HbsAg = small hepatitis B surface antigen; S-HDAg = short hepatitis D antigen; ss(+) RNA = single positive-stranded RNA; SVPs = subviral particles; UTR = untranslated region; VPg = viral protein genome-linked.

HAV belongs to the Hepatoviruses of the Picornaviridae family and has a single-stranded RNA genome (schematically shown in Figure 1). This encodes a single polyprotein, which is translated 5′cap-independently under the control of the internal ribosomal entry site (IRES) in the 5′UTR, and co-translationally cleaved into between eight and ten viral proteins [16,18]. The 5′UTR is linked to a viral protein (VPg) and the 3′UTR ends with a poly(A) tail. The capsid antigen is highly conserved and, unlike other RNA viruses, there is only one HAV serotype. The virion exists as a non-enveloped form in the stool and as a quasi-enveloped virion (eHAV) in the blood of infected patients. Membranous eHAV vesicles, secreted by hepatocytes, contain the capsid with the viral genome, but exhibit no viral proteins on their surface [17,18]. Of the seven HAV genotypes, four (I, II, III and VII) are of human origin, with I and III being the most common, and three genotypes (IV, V, VI) originate from Simians [16].

Infected children are mostly asymptomatic, while the symptoms of acute hepatitis, such as loss of appetite, fever, headache, nausea, diarrhea, anorexia, myalgia, dark urine and jaundice, appear in adults after a long incubation period (14–28 days) and are sometimes severe or even lethal [17]. Viremia and stool excretion may last longer in HIV-infected people, increasing the potential risk of transmission to others. However, the most efficient prevention against HAV-induced hepatitis is HAV vaccination [16].

### 3.2. Hepatitis B (HBV)

Infection with the highly contagious hepatitis B virus (HBV) can lead to acute or chronic hepatitis and increases the risk of liver cancer by a factor of 20 [19]. The majority of HBV is transmitted perinatally or sexually through contact with the blood or mucous membrane of an infected person. Transmission by close contact, for example, between children, is also possible [13].

HBV is a member of the Hepadnaviridae family and exclusively infects liver cells. The infectious form is called Dane particle and measures 42 nm in diameter. In addition to the virion, large amounts of non-infectious subviral particles (SVPs) only containing envelope proteins are secreted (for schematic overview, see Figure 1). Their function has not yet been clarified, but a role in immune evasion is suspected [15]. The virus is wrapped in a lipoprotein envelope and has a nucleocapsid containing double-stranded DNA with an associated polymerase. Virus replication is subject to a high mutation rate and works via reverse transcription of an RNA intermediate product [6]. Important HBV markers include the hepatitis B surface antigen (HBsAg), the hepatitis B core antigen (HBcAg) forming the nucleocapsid, a peripherally secreted protein (HBeAg) and the genomic HBV DNA [12] (schematically depicted in Figure 1).

Depending on the infectious phase, certain markers can be detected in the patient. During the initial immune tolerance phase, there are high levels of HBV DNA and secreted HBeAg in the blood, indicating active viral replication. This phase is often absent or very brief during the infection of adult patients. When mutated, the viruses are no longer able to express HBeAg, which leads to a significantly lower replication rate and a lack of HBeAg in the blood of the patients [12]. Nevertheless, chronic hepatitis B can occur in both HBeAg-positive and HBeAg-negative infected people [20]. In the second immune clearance phase, the immune system gains control over the virus and the concentration of HBV DNA in the blood begins to decrease. A prolongation of this phase could result in rapid disease progression and a high incidence of cirrhosis. However, most patients become inactive carriers with low DNA concentrations and detectable anti-HBe antibodies, while HBeAg disappears. If the virus restarts to replicate in this third phase, HBV DNA can be detected again in blood, and a subsequent inflammatory reaction can lead to liver damage or even to cirrhosis [12].

Not even the virus, but the host’s immune system is responsible for the hepatocellular damage. Only 5% of infected adults, but 90% of infants, develop chronic hepatitis. Evasion of the virus-specific immune response leads to the persistence of the virus and chronic hepatitis. The suppression of natural killer (NK) cells and the expression of immunosuppressive immune cell inhibitors prevent an effective immune response against HBV. Therefore, novel cancer immunotherapies focus on the inhibitory mechanisms of immune regulation. An additional obstacle in the therapy against HBV is a stable, covalently closed circular DNA (cccDNA), which remains in the host cell nucleus in the form of a mini chromosome or episome, even after the virus has been successfully eradicated. During infection, HBV-DNA is translocated to the nucleus of infected hepatocytes and is converted into cccDNA. It serves as a genomic template for viral mRNA synthesis, leading to a later flare-up of the infection. This cccDNA cannot be depleted neither by the immune system nor by drugs [6,19].

Around 257 million people worldwide are affected by chronic hepatitis B; however, the majority does not notice the infection. The available vaccine made it possible to successfully decrease the infection rate, but is ineffective against existing infections [19]. Virus replication can be controlled with antiviral treatments using immunomodulators such as interferon alpha or nucleoside analogues that block reverse transcriptase, but they are associated with many adverse effects and a cure is currently not possible [15,19]. In most cases, ongoing treatment is required, which is often complicated and requires an infrastructure [14]. However, treatment can drastically reduce the risk of HCC [12].

Ten characterized HBV genotypes (A to J) and several subgenotypes are geographically dispersed. They can provide information about possible mutation patterns and different clinical results, such as the delayed seroconversion of HBeAg or virus suppression. Recently, mutations in the surface gene encoding HBsAg resulted in infections of already-vaccinated children in Southeast Asia [6]. Additionally, the genotype can also be an important influencing factor in antiviral therapy [20].

### 3.3. Hepatitis C (HCV)

The hepatitis C virus (HCV) is an enveloped virus with a single-stranded RNA belonging to the Flaviviridae family and it primarily infects hepatocytes [21] (for a schematic overview, see Figure 1). The HCV genome codes for the HCV polyprotein, which is further processed into structural proteins, such as the HCV core protein, forming a nucleocapsid, and the two envelope proteins E1 and E2, located in the host-derived lipid envelope, as well as functional proteins for post-translational processing, HCV replication, RNA synthesis, virion assembly, and virion release [22,23].

As in other RNA viruses, the 5′untranslated region of the HCV genome contains a conserved internal ribosomal entry site (IRES), consisting of three loops and a pseudoknot, which allows for recognition by the ribosomes and a cap-independent translation of the viral proteins. Natural mutations in this region are associated with differences in translational efficiency [24]. The HCV genome is very diverse and classified into seven genotypes with several subtypes [25]. Glycoprotein E2 displays the highest diversity, considerably affecting immune response because neutralizing antibodies are mainly directed against epitopes on E2 or E1E2 heterodimers [23].

HCV does not stably integrate into the host cell like HBV, but has the ability to evade the immune system through the formation of viral variants or quasi-species within the same host. These variants arise from the RNA-dependent RNA polymerase’s lack of proofreading ability during high-rate replication [12,22]. The host’s immune system plays a crucial role in both eradication and progression of the infection. Only a small proportion of patients recover after HCV infection due to their strong immunity [26]. If the infection has not been completely eradicated by a strong initial immune response, persistent pro-inflammatory mechanisms and a weak or unspecific T cell response can lead to persistent liver inflammation, macrophage activation and pro-fibrinogenic processes [25]. In addition, mechanisms such as defective antigen presentation, T cell degradation by the upregulation of T cell depletion markers and an increased activity of regulatory T cells can lead to inefficiency of the immune system and loss of antiviral function [22,26].

In contrast to HBV-infected individuals, a high proportion of people (74–86%) with HCV infection develop persistent, asymptomatic viremia, of which 15–20% develop asymptomatic cirrhosis after up to 20 years and/or HCC after approx. 10 years [12,13]. Increased blood concentrations of transaminases, bilirubin, serum globulin and albumin, a low platelet count and liver stiffness display the extent of liver damage [26]. Age at the time of infection, male sex, obesity, coexisting diseases such as hepatic steatosis or HIV infection, and harmful external factors such as smoking or increased alcohol consumption are suspected to be influencing parameters [13,22].

Liver damage resulting from HCV is the main driver of the increasing demand for liver transplants [21]. Since there is neither a vaccine nor an effective therapy against HCV at present, 75% of liver transplant recipients develop a new liver infection after 6 months, leading to an even faster progression of liver disease [12,21]. This reinfection of the transplant suggests extra-hepatic replication of the virus and is associated with the occurrence of quasispecies [21].

Although HCV mainly infects hepatocytes, HCV-RNA has been detected in peripheral blood cells, cerebrospinal fluid and in the brain of patients with chronic infection [21,24]. Regardless of liver disease progress or treatment, fatigue, malesia, depression, and cognitive impairment are among the most common neuropsychiatric disorders in chronic HCV patients [27]. Recent studies have reported changes in brain metabolism and cognitive dysfunction in these patients [21]. Additionally, the interaction between HCV and diverse metabolic processes leads to alterations in glucose and lipid metabolism. By activation of the AKT/mTOR signaling pathway, downregulation of the glucose transporter 2 and impairment of the phosphorylation of AKT, the insulin signaling pathway, is inhibited. Together with an upregulated glucose production in the liver via the FoxO1-dependent signaling pathway, this leads to an increased prevalence of type 2 diabetes mellitus in chronically infected patients [26]. In addition, activated mTOR signaling seems to promote chronic infection by inhibiting apoptosis and advancing hepatocyte growth. Although mTOR inhibitors are able to decelerate disease progression in HCV-positive liver recipients, a direct molecular relation between mTOR signaling and HCV replication and the molecular relation between the two requires further research [2,28]. One function of mTOR is to transcriptionally control the interferon-mediated initiation of the translation of interferon-stimulated genes (ISGs), which are, in turn, responsible for interferon gamma (IFNγ) immune responses. Interferons are cytokines that are responsible for immunomodulation, growth-inhibition and cytocytosis [29]. As downstream targets of mTOR signaling, eIFs are responsible for both cap-dependent and IRES-dependent protein translation. In addition to pathogen recognition receptors, protein kinase R (PKR) is able to sense intracellular viral dsRNA and homodimerization, resulting in the general suppression of mRNA translation. While, under normal circumstances, eIF2 is responsible for initiating translation by recruiting Met-tRNA to the 40S ribosomal subunit, eIF2α is phosphorylated in order to block cap-dependent protein translation under stress conditions. In the case of HCV infection, it is hypothesized that PKR activation leads to a translation attenuation of ISGs, whereas HCV mRNA translation is not affected by the phosphorylation status of eIF2. Indeed, PKR activation acts pro-virally in HCV infection [30,31]. One of these ISGs, ISG56 or p56, acts in the suppression of protein synthesis under viral-induced stress conditions by binding to the eIF3e subunit. This has been shown to cause destabilization of the ternary complex comprising eIF2, GTP and Met-tRNA, and thereby functionally inhibiting cellular translation [32,33]. However, in HCV infection, a stress-mediated mRNA translation via an alternative tRNA-binding protein, eIF2A, has been shown. eIF2A works by direct binding to IRES and, at the same time, by activating PKR to phosphorylate eIF2α [31].

A few years ago, antiviral therapy with a combination of peginterferon and ribavirin represented the gold standard, but the long-term suppression of viral replication only succeeded in around 50% of patients and was heavily dependent on the viral genotype [22]. In addition, many patients experienced side effects, such as depression, cytoreduction and hemolytic anemia, during or after treatment, which mainly occurred due to the interferon-based active ingredient [26]. At present, therapy with direct-acting antiviral (DAA) drugs as specific inhibitors of viral proteins leads to a sustained virological response (SVR), which means that HCV-RNA remains undetectable 3–6 months after the treatment. In patients with chronic hepatitis, this can improve liver inflammation, liver lesions and cognitive dysfunction, even in those who had already developed cirrhosis [25,26].

### 3.4. Hepatitis D (HDV)

HDV infection only occurs as a co-infection or superinfection among people already infected with HBV, most frequently in intravenous drug users. In patients with HBV/HDV superinfection, the HBV-DNA levels are lower in both HBeAg-negative and HbeAg-positive patients, but chronic hepatitis D seems to be more aggressive than other forms of viral hepatitis. A faster progression of liver damage and, therefore, increased morbidity and mortality rates and an even higher risk of HCC can be observed [12,34]. Of the eight highly heterogeneous genotypes, seven are regionally dispersed, while genotype 1 is ubiquitous [22].

HDV is one of the smallest human pathogens and has a single-stranded, negative-sense circular RNA that encodes only one viral protein in two forms, a small (S-HDAg) and a large (L-HDAg) viral antigen for replication and virus assembly [35,36] (for a schematic overview, see Figure 1). Due to the intramolecular base pairing, the genomic RNA displays a rod-like structure and is amplified within the host’s nucleus via the antigenome, a complementary RNA strand [36,37]. By the association of the genome with S-HDAg and L-HDAg, a ribonucleoprotein (RNP) is formed and a higher degree of order is achieved, which is essential for nuclear trafficking and virus assembly [34]. For successful replication, which is HBV-independent, the virus needs important enzymes from the host, such as polymerase II and polymerase I, because an RNA-dependent RNA polymerase is absent. How the host’s DNA-dependent polymerase is manipulated to switch to an RNA template has not yet been clarified but S-HDAg might be crucial to this process [36]. HBsAg envelope proteins of the HBV virus are embedded in an ER-derived lipid vesicle in order to form virus particles that can infect hepatocytes via the HBV receptor [22,34] (schematically shown in Figure 1). The development of an antiviral therapy against HDV is rather difficult and innovative therapies aim at the entry into liver cells. HBV drugs acting on HBsAg are also able to suppress the production of HDV virions [35]. As a prophylactic measure, HBV vaccination not only prevents HBV infection, but is also highly effective against HBV-dependent HDV [37].

### 3.5. Hepatitis E (HEV)

Infection causes an enteric, acute hepatitis that primarily occurs in developing countries. It was thought to be rare in developed countries and to only occur in travelers or in patients with a weakened immune system [38]. At present, the increasing rate of HEV infection is the most common cause of acute viral hepatitis in numerous European countries [39]. A total of 15% of patients with HEV infection develop hepatic or extrahepatic complications. Immunosuppressed or immunocompromised patients with organ transplants, HIV infections or hematological malignancies are prone to quickly developing chronic hepatitis, liver fibrosis, cirrhosis and subsequent liver failure [38].

HEV is a small, envelope-free virus that replicates in the liver and belongs to the Hepeviridae family (schematically depicted in Figure 1). The three open reading frames (ORFs) on a single-stranded, positive-sense RNA encode an enzymatic polyprotein, including functional proteins for replication, a structural capsid protein, and a cytoskeleton-associated phosphoprotein. Among these, the structural proteins provide vulnerable epitopes for the immune system. Recently, a key mechanism was discovered within the translation initiation of the HEV genome in the mammalian host. The use of the IRES structures is required for other viruses, as cap-independent translation is inhibited by Interferon-induced protein with tetratricopeptide repeat 1 (IFIT1). This protein, in turn, is actively repressed by HEV RNA-dependent RNA polymerase (RdRp), thus enabling translation initiation [40]. It is assumed that similar mechanisms to those already described for HCV prevent an adequate immune response in immunosuppressed patients, and that persistent pro-inflammatory mechanisms and a weak or unspecific T cell response lead to chronic HEV hepatitis [38]. In addition, membrane-associated circulating virions have been detected in the blood. In contrast to the naked virions found in the liver and in feces, membrane-associated circulating virions possess a quasi-envelope (eHEV), and thus remain invisible to anti-capsid and anti-phospholipid antibodies. In order to achieve this, the virus makes use of the host cell’s endosomal sorting complex required for transport (ESCRT) machinery [41].

Eight genotypes have been classified; among these, HEV1-HEV4 are the most common. HEV1 and HEV2 cause major epidemic outbreaks in developing countries through contaminated water as well as severe hepatitis in pregnant women and children. HEV3 and HEV4 are responsible for sporadic cases due to zoonotic transmission by fecal contamination of water and the consumption of contaminated meat or milk [39]. The development towards chronic hepatitis has only been observed in immunodeficient patients with HEV3 infection to date [38].

Although therapy using ribavirin and interferon-alpha is available, this is not suitable for pregnant patients or transplant recipients. For these groups and ribavirin-resistant HEV-infected patients, novel and safe antiviral compounds should be found [39].

## 4. Alcohol-Induced Liver Disease

**Figure 2:**
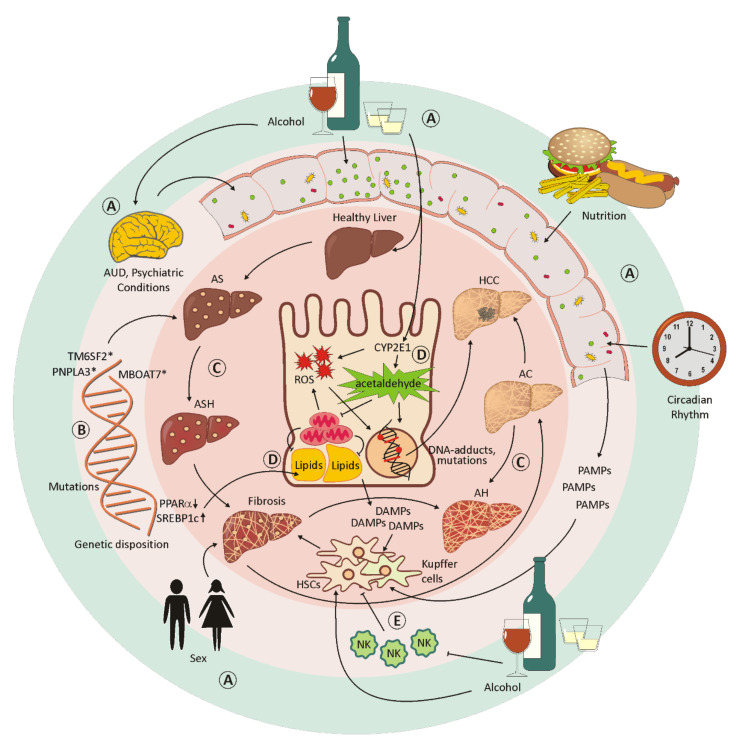
Impact of alcohol overconsumption on hepatic processes. (**A**) Chronic alcohol consumption alters the intestinal microbial community, which is also dependent on external influences, such as diet, lifestyle (circadian rhythm) and psychological conditions. The latter include AUD, depression and anxiety disorders, which manifest due to alcohol overconsumption. In addition to extensive alcohol consumption, the gender of the patient is an essential factor in the development of ALD. Women develop ALD within a shorter time, and with lower doses of alcohol. (**B**) In addition, genetic factors determine the risk and severity of ALD. Mutations in genes encoding PNPLA3, TM6SF2 or MBOAT7 lead to an impaired fatty acid metabolism and promote the development of liver steatosis. (**C**) The toxic effects of chronic alcohol overconsumption lead to AS by the accumulation of fatty acids in hepatocytes, ASH by an inflammatory reaction, fibrosis by fibrinogenic processes, and AC by the impairment of blood circulation and loss of hepatocyte function. In some cases, this leads to AH by acute-on-chronic liver inflammation or HCC by disrupted DNA repair and altered gene regulation. (**D**) In the alternative CYP2E1-mediated pathway, ethanol is metabolized to the extremely toxic and carcinogenic acetaldehyde, causing structural and functional damage in the cell. Reduced formation of ATP in mitochondria leads to the production of ROS. Together with acetaldehyde, this forms DNA-adducts, thereby increasing the mutation rate and risk of developing HCC. The progressive accumulation of fat is a result of lipid metabolism malfunction caused by the disruption of the mitochondrial β-oxidation of fatty acids, upregulation of lipogenic factors such as SREBP1c, and the inhibition of PPARα, involved in the oxidation and transport of fatty acids. (**E**) Liver cell damage activates HSCs, producing extracellular matrix. Fibrous tissue formation is triggered by pro-fibrinogenic cytokines and chemokines from liver macrophages, as well as by direct stimulation in the presence of alcohol, acetaldehyde and ROS. NK cells normally counteract the fibrinogenic effect of HSCs, but are blocked by increased alcohol consumption. Dying hepatocytes release DAMPs which, together with gut-derived PAMPs, lead to the activation of Kupffer cells, triggering an inflammatory reaction. Abbreviations (in alphabetical order): AC = alcoholic cirrhosis; AH = alcoholic hepatitis; AS = alcoholic steatosis; ASH = alcoholic steatohepatitis; AUD = alcohol use disorder; CYP2E1 = cytochrome P450 2E1; DAMPs = damage-associated molecular patterns; HCC = hepatocellular carcinoma; HSCs = hepatic stellate cells; MBOAT7 = membrane-bound O-acyltransferase domain-containing protein 7; NK = natural killer cell; PAMPs = pathogen-associated molecular patterns; PNPLA3 = patatin-like phospholipase domain-containing protein 3; PPARα = peroxisome proliferator-activated receptor-α; ROS = Reactive Oxygen Species; SREBP1c = sterol regulatory element binding protein-1c; TM6SF2 = transmembrane 6 superfamily member 2.

In most cases, chronic alcohol abuse leads to severe physical and psychological damage. Alcohol-induced liver disease (ALD) is associated with considerable mortality and includes abnormalities such as fatty liver (steatosis), steatohepatitis (ASH), alcoholic hepatitis (AH), progressive fibrosis and, finally, alcoholic cirrhosis (AC) and/or hepatocellular carcinoma (HCC) [42,43]. Chronic alcoholism also contributes to cancers of the oropharynx, the esophagus, the intestine and the female breast, and to cardiovascular diseases and neuropsychiatric disorders such as epilepsy and depressive disorders [44,45]. Although alcoholism is considered a preventable cause of disease, around 3.3 million deaths are caused by alcohol-related organ damage, traffic accidents and violence worldwide [46]. A strong component of this condition is alcohol-use disorder (AUD). This behavioral condition is characterized by a tolerance to the psychotropic effects and ignorance of the harmful effects of alcohol consumption. It is usually associated with neural impairment, accidents, injuries and psychological disorders such as anxiety and depression [44,46]. Alcohol abuse not only has far-reaching effects on the lives of patients, it also affects their social environment and the health system. Whereas the spectrum of alcoholic liver diseases ranges from asymptomatic liver steatosis, fibrosis and cirrhosis to alcoholic hepatitis, the severity and progression of the disease depends on the extent of alcohol consumption, the genetic disposition and the gender of the patient [45,46]. Women seem to be more sensitive to increased alcohol consumption and develop ALD within a shorter time, and with lower doses of alcohol, compared to men [46]. However, since only about 15–20% of chronic heavy drinkers develop alcohol-associated liver diseases, a complex interplay between environmental and genetic risk factors is assumed [47]. Coexisting aspects, such as poor diet, inactive lifestyle, depression, and changes in the circadian rhythm, are considerable influencing factors [45,47,48]. In addition, pre-existing conditions such as obesity, the intake of certain drugs or vitamins, or illnesses such as hepatitis B and C virus infections, hereditary hemachromatosis, α1-antitrypsin deficiency, and non-alcoholic steatohepatitis (NASH) may contribute to a more severe progression of ALD [45]. An overview of the extrinsic and intrinsic factors that affect the course of liver disease is schematically depicted in Figure 2A–E. 

In more recent studies, the interactions between liver and intestinal microbiota have been investigated. The composition of the microbial community varies not only individually, but also over a person’s lifespan, and is more dependent on external influences, such as diet and lifestyle, than on the host genome [45]. Alcohol has been shown to negatively influence both the composition of the microbiota and the intestinal barrier [48,49]. Microbial changes in the intestinal community could lead to a distinct bacterial or fungal dysbiosis. An increased endotoxin production by potentially pathogenic microorganisms contributes to the development of diseases such as liver cirrhosis, inflammatory bowel disease (IBD), Parkinson’s disease, autism and Clostridium difficile infection [45,50]. In patients with chronic alcohol abuse, an increased production of bile acid leads to a higher intestinal permeability and, subsequently, to elevated levels of metabolic products and endotoxins in the serum. The intestinal barrier is a dynamic structure functionally composed of intestinal epithelial cells connected by tight junctions, a protective layer including glycocalix, muscus, lysozymes, and defensins, gut immune cells and associated microorganisms [51]. The direct and indirect effects of alcohol binge and chronic alcohol abuse lead to cellular damage to intestinal epithelial cells, transcriptional down-regulation of cell junction proteins involved in tight junctions such as occludin and zona-occludens-1 (ZO-1), and tight junction disruption by the highly toxic metabolite acetaldehyde. Upon loss of mucosal integrity, endotoxin and bacterial nucleic acid levels in the serum increase [52] and an elevated translocation of bacteria and their metabolites to the liver affects bile acid metabolism and promotes inflammation [53]. Due to its close connection to the intestine, the liver is directly affected by the metabolic products of the intestinal microorganisms [45] (schematically displayed in Figure 2A).

Many studies have shown that genetic components play a role in both AUD and ALD [46,48] (schematically depicted in Figure 2B). Patatin-like phospholipase domain-containing protein 3 (PNPLA3), transmembrane 6 superfamily member 2 (TM6SF2) and membrane-bound O-acyltransferase domain-containing protein 7 (MBOAT7) are important genetic factors that determine the risk and severity of ALD [54]. PNPLA3 is a lipase, involved in the hydrolysis of triacylglycerol molecules in adipocytes and considered a risk factor for non-alcoholic fatty liver disease (NAFLD) and HCC [47,48]. A point mutation in TM6SF2 could lead to an accumulation of liver fat due to a defect associated with the secretion of very-low-density lipoproteins, and mutated MBOAT7 results in a disorder of the acetylation of phosphatidylinositol. However, it is not clear whether the latter leads to an accumulation of liver fat [46,47]. In addition, polymorphisms in genes acting in inflammatory processes could have an impact on the course and severity of alcoholic liver disease. Such inflammation mediators include TNF, IL-1, endotoxin response genes and oxidative stress-response genes [46].

The toxic effects of ethanol are mainly related to its metabolism [42]. The ability to metabolize ethanol is altered by duration and the alcohol intake, leading to an increasing toxicity in the liver. A small percentage of the absorbed ethanol is already metabolized in the stomach. This is supposed to relieve the liver, but does not work very efficiently for women or when consuming alcohol on an empty stomach [48]. However, the principal part of alcohol is oxidized in the liver cells by the alcohol dehydrogenase to acetaldehyde, and further to acetate/acetyl-CoA [42]. Since the cytosolic alcohol dehydrogenase cannot be upregulated, it is only able to metabolize small quantities of ethanol. In case of chronic overconsumption, the microsomal ethanol-oxidizing system (MEOS) is primed for alcohol oxidation, which is an alternative, cytochrome P450 2E1 (CYP2E1)-mediated pathway [42,44,46]. Hepatic CYP2E1 can also be induced by other conditions or components in the energy metabolism such as obesity and high-fat diet, hunger and associated ketone bodies, insulin and glucagon in combination with diabetes [42]. CYP2E1 is located in the microsomes of hepatocytes, can be upregulated by a factor of from 10 to 20 in heavy drinkers, and metabolizes ethanol to acetaldehyde in the presence of oxygen and NAPDH [44,46]. However, acetaldehyde is extremely toxic and carcinogenic. By binding to proteins, it causes structural and functional alterations in the cell and is responsible for the formation of neoantigens. The function of microtubules is compromised, which leads to a malfunction of intracellular protein transport, impaired excretion and, therefore, to a swelling of the liver cells. The damage in mitochondria results in a reduced outcome of ATP in the respiratory chain and the production of reactive oxygen species (ROS) [42,46,48]. The enzyme acetaldehyde dehydrogenase, which is responsible for the metabolism of acetaldehyde into acetate, is localized in the mitochondria and also impaired in its function. Acetaldehyde is able to form DNA-adducts and crosslinks between DNA strands, thereby increasing the mutation rate with a concomitant inhibition of DNA repair enzymes. The production of ROS has similar toxic effects, resulting in both a structural and functional impairment of proteins and the formation of carcinogenic DNA-adducts [46] (see Figure 2D).

Almost all heavy alcoholics initially experience a progressive accumulation of fat as a result of a malfunction in lipid metabolism leading to an alcohol-induced hepatic steatosis [44,48]. Alcohol triggers the accumulation of fat in the liver through several different mechanisms. Firstly, elevated levels of reduced NAD (NADH) disrupt the mitochondrial β-oxidation of fatty acids. Secondly, alcohol consumption increases the expression of SREBP1c, a lipogenic transcription factor, leading to the accumulation of more fat. Thirdly, alcohol overconsumption inactivates the nuclear hormone receptor peroxisome proliferator-activated receptor-α (PPARα), employed in fatty acid transport and oxidation [46]. As a result, dying hepatocytes release damage-associated molecular patterns (DAMPs) which, together with gut-derived pathogen-associated molecular patterns (PAMPs), inflate inflammatory processes in the liver [44,46]. Lipopolysaccharides from gram-negative bacteria reach the Kupffer cells via the portal vein circulation and activate them by the toll-like receptor 4 (TLR4). Thereby, an inflammatory reaction is initiated and sustained by the release of pro-inflammatory cytokines and chemokines [48]. An additional activation of the immune system is induced by the formation of neo-antigens by acetaldehyde and ROS [46]. Alcohol-induced steatohepatitis (ASH) is histologically characterized by steatosis, lobular inflammation, Mallory–Denk Body (MDB) formation, and the ballooning of damaged liver cells, further advancing to fibrosis and cirrhosis [55,56].

One of the earliest fibrotic changes in the liver is collagen deposition around terminal hepatic veins [56]. The liver cell damage caused by chronic alcohol consumption subsequently stimulates the activation of wound-healing mechanisms. Activated hepatic stellate cells (HSCs) are responsible for this, although portal fibroblasts and bone-marrow-derived myofibroblasts are also involved to a lesser extent [44,46]. During this process, HSCs transdifferentiate into the extracellular matrix, producing myofibroblasts [56]. The abnormal accumulation of fibrotic tissue is triggered by pro-fibrinogenic cytokines and chemokines from liver macrophages, as well as by direct stimulation by alcohol, acetaldehyde and ROS [44,46]. Natural killer (NK) cells are normally able to counteract the fibrinogenic effect of HSCs, but increased alcohol consumption blocks NK cells [46,56] (schematically displayed in Figure 2E). Histologically, alcoholic steatohepatitis is depicted by different degrees of steatosis, by inflammatory infiltrates composed of polymorphonuclear (PMN) cells predominant in the lobules, by ballooning of damaged liver cells, by necrosis, by Mallory-Denk inclusion bodies and pericellular and perisinusial fibrosis meshworks [44,46].

When the liver fibrosis further advances, the structure of the liver massively changes (schematically displayed in Figure 2C). In liver cirrhosis, unhindered blood flow is no longer possible and liver function is severely impaired by the loss of hepatocytes [46]. The liver is unable to regenerate. A massive proliferation of liver precursor cells (LPCs) in regenerative parenchymal nodes takes place, but LPCs are not able to differentiate into mature hepatocytes. Overgrowing fibrotic septa lead to the development of portal hypertension [44,57]. In addition, complications such as variceal bleeding, hepatic encephalopathy, ascites, and hepatorenal syndrome are responsible for an increase in mortality in these patients [57].

Alcoholic cirrhosis can progress into alcohol-associated hepatocellular carcinoma (HCC) under certain pathophysiological conditions. Thereby, the previously mentioned mutagenic processes triggered by acetaldehyde and ROS are of considerable importance. Apart from stable DNA-adduct formation, point mutations and sister chromatid exchange, DNA repair is insufficient and pro-oncogenes could be activated into oncogenes by induced CYP2E1 [46]. In addition, changes in DNA methylation due to increased alcohol consumption lead to epigenetic changes. The survival rate of HCC patients depends on the epigenetic silencing of tumor-suppressor genes and the activation of oncogenes by hypomethylation [58].

As patients with early ALD usually show no symptoms and keep their alcohol abuse private, diagnosis is often only based on clinical suspicion, laboratory tests and external signs such as muscle-wasting, malnutrition or peripheral neuropathy. Symptoms such as rapidly progressing jaundice, fever, abdominal pain and weight loss are associated with ASH, while patients with alcoholic hepatitis (AH) display a systemic, inflammatory syndrome with tachycardia, leukocytosis and elevated levels of C-reactive protein and procalcitonin [44]. 

Liver transplantation is often the only long-term treatment option for patients with severe end-stage liver disease. Since many countries require at least six months of abstinence before surgery and there is a long waiting list for liver transplants, this treatment is usually not an alternative for patients with severe chronic alcohol abuse and alcohol-use disorder [43,46]. This waiting time is intended to enable the liver function to be restored and to reduce the risk of relapse after transplantation [43]. Due to this rule, patients with acute chronic alcoholic hepatitis (AH) (see Figure 2C) are unsuitable for a transplant, since 20–50% die within 3 months if they do not respond to corticosteroids [44,56]. 

In a pilot study, nasogastric fecal microbial transplantations (FMTs) in patients with AH were able to improve disease, dysbiosis, and survival rate [49]. Non-invasive therapies could be of enormous importance for the future. Such therapeutic approaches could include the modification of the intestinal microbiota to both strengthen the intestinal barrier and alleviate the psychological effects of alcoholism, thus preventing alcohol-related liver changes [45,48].

Even if the direct effect of alcohol on protein translation is not known at present, cellular stress can lead to an unfolded protein response (UPR) inside the endoplasmic reticulum (ER). The ER-located, stress-inducible protein kinase PERK phosphorylates the alpha subunit of eIF2 in response to unfolded proteins that are sensed inside the ER. This results in an attenuation of protein translation to avoid the accumulation of unfolded or misfolded proteins and hepatocyte apoptosis [10].

Another disease caused by an unhealthy lifestyle, sharing many pathogenic and histological features with ALD, is the NAFLD [55]. 

## 5. Non-Alcoholic Fatty Liver Disease

**Figure 3:**
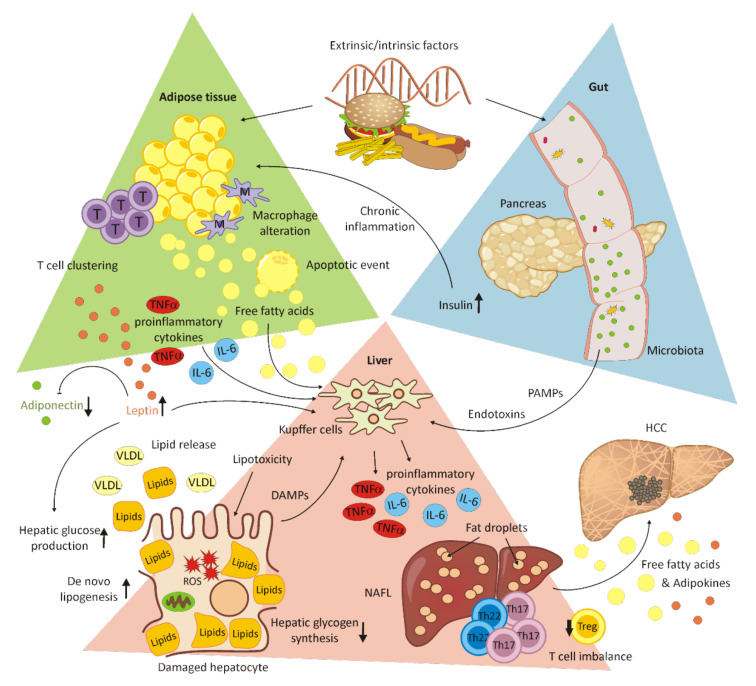
Chronic inflammation in adipose tissue and its effects on the liver. Adipose tissue. Chronic inflammation of the adipose tissue results in T cell clustering and ATM alteration results in characteristic apoptotic events in adipocytes and elevated levels of circulating free fatty acids, which can directly stimulate Kupffer cells. Leptin is involved in glucose homeostasis and immune response. High levels of leptin in obese individuals, together with low levels of adiponectin, contribute to disease progression to steatosis and fibrosis. Gut/Pamcreas. Hyperinsulinemia induces chronic inflammation in adipose tissue, while gut-derived endotoxins and PAMPs, together with DAMPs of dying hepatocytes, activate Kupffer cells in the liver, which secrete proinflammatory cytokines. Liver. Lipid overload in the liver triggers hepatocyte cell death, while DAMPs are released and inflate inflammatory processes in the liver. Kupffer cells and monocyte-derived macrophages (not shown) promote liver inflammation by the secretion of proinflammatory cytokines. The proinflammatory cytokines TNFα and IL-6, as well as adipose tissue-derived adipokines, are known to induce T cell imbalance in favor of proinflammatory Th17/Th22 T cells versus Treg cells. Abbreviations (in alphabetical order): ATM = adipose tissue macrophage; DAMPs = damage-associated molecular patterns; HCC = hepatocellular carcinoma; IL-6 = interleukin 6; M = macrophages; NAFL = non-alcoholic fatty liver; PAMPs = pathogen-associated molecular patterns; T = T cells; Th17 = Type 17 T helper (Th17) cell; Th22 = Type 22 T helper (Th22) cell; TNFα = tumor necrosis factor alpha; Treg = regulatory T cells; VLDL = very-low-density lipoproteins.

NAFLD is the most common chronic liver disease [59,60] with an incidence ranging from 6.3% to 33% worldwide, from 5% to 18% in Asia [61,62] and from 20.3% (women) to 33.4% (men) [59] in Europe, depending on the assessment method [63,64]. A rising trend is found in Europe, the United States, the Middle East and Asia [64,65,66,67,68,69,70]. An NAFLD prevalence of 9.6% was revealed after children were autopsied and, among obese children, 38% were diagnosed with NAFLD [71,72]. NAFLD is a term that includes non-alcoholic steatohepatitis (NASH) with inflammation, hepatocellular injury, fibrosis, cirrhosis derived from fibrosis [60] and non-alcoholic fatty liver (NAFL) [61]. A total of 50% of obese individuals were diagnosed with NASH in combination with the comorbidity diabetes mellitus (DM) [73]. NAFLD is diagnosed when a macrovesicular steatosis is found in 5% or more hepatocytes without alcohol as a cause [74,75,76]. The risk factors of NAFLD are obesity, diabetes mellitus and dyslipidemia, insulin resistance and ethnicity as primary risk factors, and sex, age, obstructive sleep apnea, polycystic ovary syndrome, drugs and toxins as secondary risk factors, with further correlations with family history [60]. The results of NAFLD are indistinguishable from alcoholic liver disease [60,77]. The primary countermeasures against NAFLD are weight loss, followed by insulin sensitizers, bariatric surgery, antioxidants, lipid-lowering drugs and liver transplantation [72]. Serum-free fatty acids are imported and processed by the liver and lipoproteins, and lipids are secreted from the liver. In NAFLD, triglyceride accumulation leads to hepatocellular damage, mainly correlating with insulin resistance. Insulin resistance has an influence on lipid oxidation and exportation [73]. The hormones produced from adipose tissue, oxidation and bacterial toxins arising from the gut can result in a second hit, leading to the transformation of steatotic liver into NAFLD [73,78] (for a schematic overview, see Figure 3). 

As described before in ALD, in tissue samples of obese NAFLD patients, elevated levels of UPR makers, indicating ER stress, have been detected. Insulin resistance has been shown to follow the phosphorylation of insulin receptor substrate 1 (IRS-1) by JNK activation linked to ER-stress. However, the roles of UPR and eIF2α phosphorylation in the development of ER-stress-associated steatosis remain unclear [10].

Insulin resistance affects glycogen storing and glucose uptake, including fatty acid delivery, triglyceride secretion and the triglyceride esterification pathway. Glycogenesis in hepatic cells is reduced. Glucose uptake decreases and leads to a decline in glucose-6-phosphate, starting from glucose, and a shift in glucose-6-phosphate towards an increase in de novo lipogenesis. Fatty acid delivery leads to a rise in CO_2_ by oxidation instead of oxidizing glucose-6-phosphate. Fatty acid delivery also promotes triglyceride esterification and very-low-density lipoprotein (VLDL) secretion. Increased amounts of glycerol and lactate provide gluconeogenesis and lead to a rise in glucose-6-phosphate. In the context of insulin resistance, glucose-6-phosphate is rarely a product of glucose [79].

Lipid metabolism disorders affect the development of steatohepatic HCC (SH-HCC). The disease is characterized by HCC in combination with steatosis and its development occurs in a hypoxic microenvironment. Under hypoxic conditions, the mTOR pathway activation, as well as lipid accumulation and upregulated hypoxia-inducible transcription factor (HIF)-2α, have been observed in NALFD-HCC patients, correlating with a poor overall survival [80]. In addition, the chronic inflammation of adipose tissue caused by hyperinsulinemia and insulin resistance in obese individuals promotes the secretion of proinflammatory cytokines, such as tumor necrosis factor alpha (TNFα) and interleukin 6 (IL-6). These cytokines activate JNK and STAT3, promoting the transcription of apoptotic-, inflammation-, proliferation-, differentiation- and angiogenesis-related genes. Both immune response and glucose homeostasis are regulated by leptin. High levels of leptin, which are typically found in obese individuals, together with low levels of adiponectin, contribute to disease progression to HCC. By the activation of JAK/STAT3 and PI-3K/Akt, activation of the mTOR pathway occurs, which has been reported in 30–40% of HCC patients. In turn, the upregulation of SOCS3 by adiponectin represses STAT3 and Akt phosphorylation. However, in obesity, this anti-inflammatory effect is reduced due to low adiponectin levels [81]. A closer view of the immune system suggests the participation of both the innate and the adaptive immune system in adipose tissue inflammation and disease progression. T cell accumulation in adipose tissue results in adipose tissue macrophage (ATM) alteration and immune cell clustering, followed by characteristic apoptotic events in adipocytes. Representative players in innate immunity, in the context of NAFLD, are Kupffer cells and monocyte-derived macrophages promoting liver inflammation by the secretion of proinflammatory cytokines. Kupffer cells are directly stimulated by circulating free fatty acids. Furthermore, NK cells and dendritic cells play a crucial role in pathogenesis [82,83]. The proinflammatory cytokines TNFα and IL-6, as well as adipose-tissue-derived adipokines, are known to induce the differentiation of proinflammatory T cell subsets. This leads to a T cell imbalance in favor of Th17/Th22 T cells compared to Treg cells [82].

## 6. Autoimmune Hepatitis

**Figure 4:**
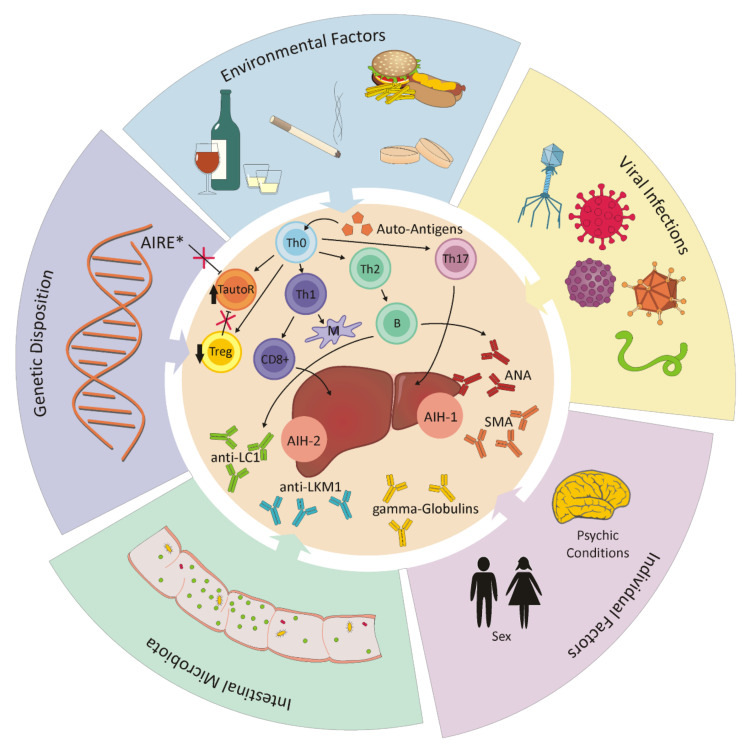
External and intrinsic factors modulating the immune system. Although the causes have not been fully revealed, environmental factors such as diet, alcohol or drug consumption and viral infections, as well as intrinsic factors such as psychic conditions, the patient’s sex, intestinal microbiota and genetic disposition, are supposed to promote the development of autoimmune hepatitis. Independently of age, females are affected 3–4 times more frequently than males. It is supposed that a failure in immune tolerance leads to a CD4^+^ and CD8^+^ T-cell-mediated immune attack against liver antigens. A decrease in Treg cells or mutation (✷) in the AIRE gene lead to a loss of the homeostasis between autoreactive T cells (TautoR) and other effector CD4+ cells and, furthermore, to the production of gamma globulins and autoantibodies such as ANA and SMA in type 1 autoimmune hepatits and anti-LC1 and anti-LKM1 in type 2 autoimmune hepatitis. Abbreviations (in alphabetical order): AIH-1 = autoimmune hepatitis type 1; AIH-2 = autoimmune hepatitis type 2; AIRE = autoimmune regulator protein; ANA = antinuclear antibodies; anti-LC1 = anti-liver-cytosol-type 1 antibodies; anti-LKM-1 = anti-liver-kidney microsome-type 1 antibodies; B = B-lymphocyte; CD8^+^ = cytotoxic T lymphocyte; M = macrophage; SMA = anti-smooth muscle cell antibodies; TautoR = autoreactive T cells; Th0 = naive T cells; Th1 = Type 1 T helper (Th1) cell; Th17 = Type 17 T helper (Th17) cell; Th2 = Type 2 T helper (Th1) cell; Treg = regulatory T cell.

Autoimmune hepatitis (AIH) histologically presents itself as chronic, and sometimes as acute, hepatitis without a known cause and without disease-specific markers [84,85]. This type of liver disease occurs in children and adults of all ages, whereas women are affected 3–4 times more frequently than men [86,87]. The causes have not been fully disclosed at present, but it is supposed that a failure in immune tolerance leads to a CD4+ and CD8+ T-cell-mediated immune attack against liver antigens, resulting in progressive inflammation and fibrosis in the liver. Both external stimuli and genetic disposition could be responsible for this malfunction [87] (schematic overview depicted in Figure 4). Autoreactive T cells also circulate in healthy people, but are controlled by immune tolerance mechanisms to avoid tissue damage. This homeostatic process is mostly maintained by regulatory T cells (Treg), which directly derive from naïve T cells and make up approximately 5–10% of all peripheral CD4+ T cells. By direct contact with target cells, they control immune responses by limiting both the proliferation and the effect of autoreactive T cells. A lower number of circulating Treg is found in AIH patients than in healthy people, which suggests a decreased ability to control CD4+ cell proliferation [88,89] (altered immune regulation depicted in Figure 4). In addition to defects in quantity and efficiency, an elevated conversion of Treg into effector cells can be observed under inflammatory conditions. With the production of pro-inflammatory cytokines, Th17 cells are also involved in autoimmunity. The tasks of Th17 cells include both the recruitment of neutrophils to sites of infection, and the immune responses against extracellular bacteria and fungi [90]. By inducing hepatocytes, they can promote differentiation into more Th17 cells. Although their role in the pathogenesis has not yet been clarified, elevated levels of Th17 cells have been observed in AIH patients. [89]. While the Th1 subpopulation of CD4^+^ effector cells conducts cellular immunity by activating cytotoxic CD8^+^ lymphocytes and macrophages, Th2 CD4^+^ cells induce B-cell-mediated antibody production. The particular cytokines of each system can cross-inhibit the other system. In case of autoimmunity, Th1 cells seem to mediate organ-associated autoimmunity such as Crohn’s disease, sarcoidosis, and acute kidney allograft rejection, while Th2 lymphocytes are allergen-specific and responsible for systemic autoimmune diseases [88,91]. Together with autoimmune hepatitis, a simultaneous extrahepatic autoimmunity is often observed. Furthermore, there is an interaction between AIH and pregnancy, where AIH usually does not occur during pregnancy, but after birth [92].

Faulty genes could inhibit the negative selection process of autoreactive immune cells, and thus allow for the detection of similar auto-antigens in the same tissue or in other organs. However, the genetic screening of patients or family members is unnecessary because AIH is a complex, polygenic disease and it is very unlikely that this disease will be passed onto the offspring. An exception is patients with a mutation in the autoimmune regulator protein (AIRE), which acts as a transcription factor and regulates the clonal extinction of autoreactive T cells in the thymus. Such patients are commonly affected by AIH, but show a recessive heredity and no female preponderance [87].

As external triggers, virus infections can promote the development of AIH. Often, the infection dates back years, which makes a retrospective identification of the pathogen impossible. Molecular mimicry by the cross-reactions of viral epitopes and certain liver antigens is conceivable. Pathogens such as measles virus, cytomegalovirus or Epstein–Barr virus could act as a disease triggers; however, the involvement of hepatitis viruses is most likely [86]. The defects in immune response could be induced by the same pathogenic trigger, which is characteristic for the host or the disease [84].

More recently, the influence of gut microbiota on the pathogenesis of AIH has been taken into consideration. An association between changes in the composition and variety of intestinal microbiota (dysbiosis) has been observed in experimental models of AIH compared to healthy volunteers. It is supposed that the reduction in anaerobic bacteria could be responsible for the increased diffusion of intestinal microorganisms into systemic circulation by reducing the intestinal permeability [89].

The symptoms of AIH are often unspecific, but the disease can also begin asymptomatically or as a severe acute hepatitis. Therefore, diagnosis must be made on the basis of histological findings and the detection of characteristic circulating autoantibodies. The serological examination of most AIH patients shows increased levels of either antinuclear antibodies (ANA), anti-smooth-muscle-cell antibodies (SMA), liver–kidney microsome-type 1 antibodies (anti-LKM-1), liver-cytosol-type 1 antibodies (anti-LC1) and increased levels of gamma globulins [93,94]. These findings indicate an activated immune system and consequential cytotoxic mechanisms. Based on the detected autoantibodies, two subtypes of the disease can be differentiated [84,87,93]. Subtype AIH-1 is characterized by the serological reactivity of ANA, SMA or both autoantibodies, and accounts for 80% of all AIH cases. The detection of anti-LKM-1 or anti-LC1 refers to subtype AIH-2, which occurs less often and mainly in pediatric patients [87,95]. Occasionally, however, there are AIH patients in which none of these autoantibodies can be detected. It might be helpful to search for other atypical autoantibodies such as pANCA, which are specific to primary sclerosing cholangitis (PSC) and inflammatory bowel disease (IBD) [86,87].

Acute autoimmune hepatitis is often difficult to diagnose due to the undetectable autoantibodies or low IgG in the serum. Therefore, for diagnosis, histological findings are very important to show plasma cell infiltrations and portal or lobular infections in the liver tissue [84,96]. 

AIH is uncommon and clinical diagnosis is often delayed due to the unspecific symptoms and lack of standardized laboratory tests [94]. Variants and mixed forms of AIH make diagnosis more difficult, as they show similarities to other potential autoimmune liver diseases, such as primary biliary cirrhosis or primary sclerosing cholangitis. Additionally, liver cell damage caused by the consumption of certain drugs can be mistaken for AIH [86]. For an accurate diagnosis, it is important to exclude other liver diseases that are similar to AIH, such as viral hepatitis or hereditary, metabolic, cholestatic or drug-induced liver damage [85]. In the elderly population of over 60 years, the diagnosis and treatment of AIH can be even more difficult due to drug interactions, additional diseases and the frequent occurrence of cirrhosis [94].

Classical histological markers for AIH are the presence of a portal inflammation with eosinophilic granulocytes, plasma cell infiltrates, mainly lymphocytes in the portal tract, emperipolesis and the rosette formation of liver cells. However, all these markers can also be detected in HCV-induced chronic hepatitis [97] and are only faintly seen, or not seen, in approximately one third of the AIH cases [95]. In other recent studies, the detection of plasma cell clusters (more than five cells) in the lobulus is described as a sensitive diagnostic marker for AIH [95,97]. In addition, interface hepatitis is considered as a marker of the AIH, albeit not a specific marker. The expansion of portal inflammation into the adjacent lobules, with damage to and the progressive loss of hepatocytes, is observed in up to 98% of AIH cases and is usually more prominent than in the interface of hepatitis from other causes [95].

## 7. HCC/mTOR/eIFs

HCC had an incidence of 905,677 new cases worldwide in 2020, when 830,180 deaths were recorded. HCC ranges as the 7th most common tumor type and the fourth most common cause of cancer-associated death. HCC is more common in males, with a female:male ratio of approximately 1:3, and patients at primary diagnosis present at the rather young age of 30–50 years [98]. The molecular pathogenesis is orchestrated by the accumulation of genetic alterations that ultimately lead to malign signaling interference in molecular pathways including receptor tyrosine kinase, Mitogene-activated protein kinase, Wnt/beta-catenin signaling, ubiquitin/proteasome degradation, hedgehog signaling, and the phosphatidylinositol 3-kinase (PI3K)/AKT/mTOR signaling pathways feature here [99].

Recent studies have shown that a quarter of all HCC cases reveal potentially actionable mutations, which are not yet implemented in clinical practice [100]. Cancer tissues are known to have exceeding proliferation rates depending on an increased protein translation. While awry mTOR signaling is an established event in HCC, the role of increased and decreased mTOR directly interacting with eIFs in cancer development has not been fully disclosed. Here, Cajal et al. provide an excellent, organ-spanning review of the carcinogenic role of mal-expressed mTOR and eIF proteins [101]. Excellent recently published reviews on mTOR in HCC include Ferrin et al., 2020 [102], Sun et al., 2021 [103], and, with a therapeutic focus and the perspectives of treating mTOR in HCC, Rebouissou and Nault, 2020 [104]. The mTOR-related eIFs still have not gained clinical awareness, which is why we refrain from going deeper into this tangent.

As outlined above, chronic infections with hepatitis viruses, especially HBV and HCV, NAFLD, NASH, ASH and, furthermore, exposure to aflatoxin B1, diabetes and obesity, are the major risk factors supporting hepatocellular carcinogenesis. The induced liver parenchyma inflammation and fibrotic deposition are secondary risk factors for the development of HCC. The individual tumor’s behavior and progression are highly influenced by the acting proteome within the cancer cells, which, in turn, is shaped by genetic predisposition, oncogene activation, and altered gene expression, including epigenetic mechanisms [105]. In carcinogenesis, a deep reprogramming of cellular metabolic processes takes place. The metabolic transformation is typically characterized by molecular pathway alterations to meet the changing demands of the tumor cells [102]. Molecular pathway alterations are associated with the development and progression of HCC are numerous. Among the affected pathways, the mechanistic target of rapamycin (mTOR) signaling is of the highest relevance and will be discussed here in more detail. 

### 7.1. mTOR Signaling

**Figure 5:**
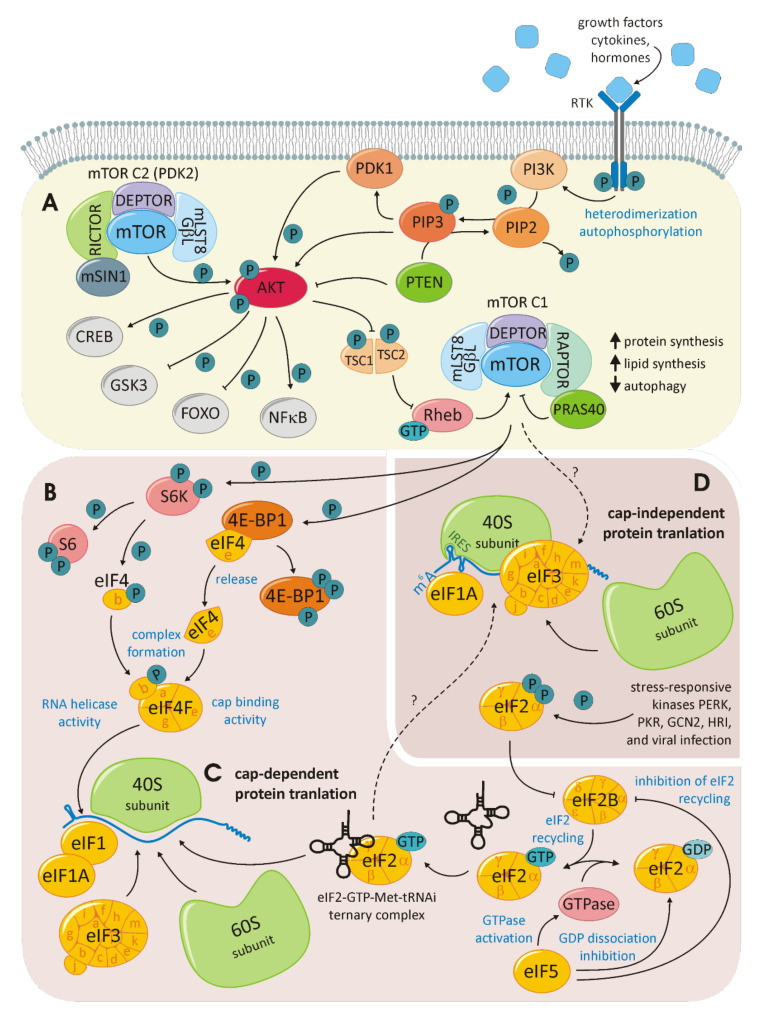
Crosstalk between mTOR and eIF signaling and possible targets for therapeutic substances. (**A**) RTK heterodimerizes after ligand binding and recruits PI3K, phosphorylating PIP2 to PIP3; PIP3 recruits PDK1 and Akt (also known as PKB); Akt is partially activated by PDK1 and fully activated by mTORC2 (PDK2) phosphorylation; the phosphatase PTEN negatively regulates Akt by dephosphorylating PIP3 to PIP2; besides mTOR, Akt activation induces downstream signaling, such as GSK-3, CREB, FOXO, and NF-kB, regulating cellular and metabolic processes, innate and adaptive immune responses, and autophagy; in addition to activated Akt, Rheb (GTPase) is required for mTORC1 activation; Akt inhibits TSC2, which inhibits Rheb, thus activating mTORC1. (**B**) mTORC1 promotes protein synthesis by phosphorylating S6K, which, in turn, phosphorylates 4E-BP1, leading to both eIF4e release and eIF4F complex formation; eIF4F complex comprises eIF4e, eIF4g, and eIF4a and represents a key component of cap-dependent protein translation; additionally, S6K activates eIF4b, a positive regulator of cap-dependent translation. (**C**) In cap-dependent translation, the mRNA 5′ cap is recognized by eIF4F complex, which shows RNA helicase and cap-binding activity; eIF4F complex recruits the 40S ribosomal subunit, eIF3, eIF1, eIF1A, eIF5, and eIF2-GTP-Met-tRNAi ternary complex to the 5′ end of the mRNA; met-tRNA affinity to eIF2 is regulated by guanine nucleotides, so affinity is high when eIF2 is bound to GTP; eIF5 hydrolyzes GTP to GDP, resulting in eIF2-GDP leaving the ribosome; eIF2B acts as GEF by the removal of eIF5 and reactivation of eIF2 with GTP; the 43S preinitiation complex scans the mRNA in a 5′-to-3′ direction and the 60S ribosomal subunit joins to form an 80S translation-competent ribosome. (**D**) Cap-dependent translation initiation is down-regulated under stress conditions or during viral infection by phosphorylation of the α subunit of eIF2 by stress-responsive kinases or viral proteins; phosphorylated eIF2α inhibits eIF2B (GEF), acting in eIF2 recycling, thus inhibiting canonical protein translation; an alternative, cap-independent mechanism of the translation of viral or cellular stress response proteins is mediated by IRES-dependent or (m^6^A)-dependent translation initiation. Abbreviations (in alphabetical order): 4E-BP1 = eukaryotic translation initiation factor 4E binding protein 1; Akt = Akt, protein kinase B; CREB = cAMP-response element-binding protein; eIF = eukaryotic translation initiation factor; FOXO = forkhead box O protein; GCN2 = general control non-derepressible-2; GEF = guanine nucleotide exchange factor; GSK-3 = glycogen synthase kinase-3; HRI = heme-regulated inhibitor; IRES = internal ribosomal entry site; Met-tRNAi = initiator methionyl transfer RNA; mTOR = mammalian target of rapamycin; NF-κB = nuclear factor κ-light-chain-enhancer of activated B cells; PDK1/2 = phosphoinositide dependent protein kinase 1/2; PERK = PKR-like ER kinase; PI3K = phosphatidylinositol 3-kinase; PIP3 = phosphatidylinositol-3,4,5-triphosphate; PKR = protein kinase double-stranded RNA-dependent; PTEN = phosphatase and tensin homolog; Rheb = Ras homolog mTORC1 binding; RTK = receptor tyrosine kinase; S6 = ribosomal protein S6; S6K = ribosomal protein S6 kinase; SREBP-1c = sterol regulatory element binding protein-1c; TSC1/2 = tuberous sclerosis complex subunit 1/2.

The mTOR anabolic pathway is highly conserved. mTOR itself is a 289-kDa, phosphatidylinositol-3-kinase (PI3K) family-related serine/threonine protein kinase that interacts with other proteins to establish two distinct complexes: mTORC1 and mTORC2 (for a schematic overview, see Figure 5A). Both complexes contain mLST8/GβL and DEPTOR. Raptor and PRAS40 are specific to mTORC1, while the proteins Rictor and mSIN1 are specific to mTORC2. While mTORC1 is associated with cell-growth control, the activity of mTORC2 controls cell survival and proliferation. A broad range of upstream signals, including growth factors, hormones and cytokines, provoke the activation of PI3K that phosphorylates and thereby activates AKT, which is the upstream activator of mTOR. For example, the insulin-like growth factor would bind the extracellular region of a receptor tyrosine kinase (RTK) in this manner, thereby activating the ligand-induced receptor dimerization, leading to kinase activation. RTKs will autophosphorylate the dimerization partner at the C-terminal tail, recruiting a variety of downstream signaling proteins containing Scr homology-2 (SH2) or phosphotyrosine-binding domains (PTBs). The PI3K contains two RTK interacting SH2 domains in its adaptor subunit. Consequently, PI3K catalyzes the generation of phosphatidylinositol-3,4,5-triphosphate from phosphatidylinositiol-4,5-bisphosphate. This reaction is competed by the tumor-suppressor phosphatase and tensin homolog (PTEN). Attracted by the newly formed phosphatidylinositol-3,4,5-triphosphate AKT is recruited to the plasma membrane, where it is phosphorylated by PDK1 and, in a second step, by mTORC2. AKT is a potent kinase phosphorylating, among others, tuberous sclerosis complex2 (TSC2) and Rheb within the mTORC1 complex. AKT may also activate mTORC1 in a TSC-independent manner. Activated mTOR phosphorylates further downstream targets involved in a lipid and nucleotide synthesis, energetic homeostasis, ribosome biogenesis, nucleotide metabolism and cell-cycle progression through mTORC1 and via mTORC2 cell proliferation and survival, cytoskeletal remodeling and cell migration [106,107]. The mTOR-dependent regulation of translation initiation is primarily executed by the phosphorylation of eukaryotic translation initiation factor 4E-binding protein 1 (4E-BP1) (as depicted in Figure 5B). Unphosphorylated 4E-BP1 is stably associated to eIF4E, thus inhibiting the eIF4F cap-binding complex formation required for the initiation of cap-dependent translation. Therefore, the phosphorylation of 4E-BP1 releases eIF4E, which joins eIF4A and eIF4G to form the eIF4F complex. mTOR interacts with another eIF complex: eIF3. eIF3 is a 13-subunit complex that is assembled in a modular manner. Different modules or eIF3 proteins interact with the ribosome, while other eIFs and translational enhancers are required to fine-tune translation initiation. As a scaffold of regulation, eIF3 also interacts with mTOR [108]. This interaction, in addition to the regulation of 4E-BPs, enables mTOR to influence cap-independent translation initiation.

The influence of mTOR goes even further and is linked to autophagy and senescence. The secretome of senescent cells is referred to as the senescence-associated secretory phenotype (SASP). Herranz et al. discovered the central role of mTOR within this field. By the downstream activation of 4E-BP, mTOR selectively controls the translation of proteins distinctly linked to SASP. Depending on the developmental stage of a tumor, SASP may impair tumor-suppressive or tumor-promoting functions. A deeper understanding of this molecular process will explain the reported beneficial effect of mTOR targeting in cancer therapy [109]. The process of cap-dependent, canonical translation requires maximal control and collaboration between acting and regulating proteins (see Figure 5C). Canonical translation initiation encompasses the phases of ribosome recruitment, mRNA scanning, initiation, elongation, and termination, as well as ribosome recycling [110]. The exploitation of alternative translation initiation mechanisms was introduced in the depiction of the gene expression modes associated with hepatitis viridae. An alternative translation initiation frequently occurs in tumor cells (schematically shown in Figure 5D). Here, IRES-dependent, N^6^-methyladenosine (m^6^A)-dependent and re-initiation-dependent translation are extensively studied. IRES-dependent translation initiation relates to the distinct secondary and tertiary structures within the mRNA that are directly bound by the 40S ribosomal subunit. This recruitment can take place unaided; however, recently, IRES trans-activating factors assisting this mechanism have been identified [111]. While the structural prerequisites for IRES-dependent translation are complex, m^6^A-dependent translation initiation relies on a basic modification. Among all posttranscriptional RNA modifications, the N^6^ methylation of Adenin is the most common. M^6^A modifications within the 5′UTR can directly recruit eIF3, triggering the formation of the 43S ribosome. This mechanism skips all cap-dependent process steps and is, therefore, independent of eIF4F [112]. Translation re-initiation occurs when the translational machinery is recruited to an upstream open reading frame (ORF) preceding the main ORF. The canonical eIF3h and the non-canonical eIF2D jointly ensure that translation is terminated within the ribosome for re-initiation on the same mRNA [113,114]. 

Hepatocellular carcinoma (HCC) is frequently associated with mTOR signaling alterations. At the same time, the common background triggering viral infections is an important feature of the disease. In HCC, 40–50% of patients upregulate the mTORC components [115]. Similarly, many of the downstream mTOR, as well as mTOR-independent eIFs, are reported to be upregulated in HCC [116]. Obviously, these molecules are all key components, with tumor characteristic hallmarks ranging from sustained proliferation to resistance to growth repression. Future studies will elucidate the fine-tuning of the various mechanisms of translation initiation, how they influence carcinogenesis and where and how they could be targeted in HCC therapy.

The most frequent accompanying infections, in 54% of HCC cases, are HBV and HCV, with local predispositions in Asia/sub-Saharan Africa and Italy/Japan, respectively. Any hepatitis viral infection interferes with the physiological signaling pathways. The resulting sustained inflammation, fibrosis and lack of hepatocyte regeneration, together with the complex modulation of the tumor microenvironment, set a stage that is hard to predict in therapy planning. 

### 7.2. HCC Therapy

Therapy options include tyrosine kinase inhibitors (TKIs), especially the multiTKI sorefenib, targeting, among others, Raf-1, Braf, VEGFR and PDGFR-beta. As a second-line therapy regorafenib, another multiTKI, was used in patients displaying sorefenib-resistance. However, due to resistance mutations in the tumor, such as the activation of MAPK/ERK signaling, cellular responses to TKIs are heterogeneous and the clinical benefit is not optimal, even with combined TKIs. The anti-proliferative and immunosuppressive effect of TKIs are only advantageous for liver transplant recipients [102]. The recent advent of immune checkpoint inhibitors (ICIs) in HCC therapy has brought about a dramatic change and set a standard of care with the combination of atezolozumab (ICI) and bevacizumab in a first-line treatment. Ding et al. have analyzed the response rates of HCC patients under immunotherapy depending on the viral status and concluded that virally infected HCC has no significant difference in therapy response to non-infected HCC [117]. Multiple HCC immunotherapy strategies were recently reviewed by Zhang et al. [118]. Future clinical studies will show how the recently reported approaches targeting eIF proteins in breast, lung, ovary or prostate cancers will also apply to HCC, as reviewed in Cajal et al. [101] and Hao et al. [119].

Rapamycin, the drug giving mTOR its name, was originally discovered as an anti-fungal metabolite and immunosuppressive agent. After being used to prevent graft-versus-host disease (FDA Approval since 1999), the pharmaceutical is also implemented in cancer therapy [120]. A list of clinical trials with the drugs Rapamycin/Sirolimus, Temsirolimus, Everolimus, AZD8055 and INK128 is available as Supplementary Tables S1 and S2, for active and completed trials, respectively [121]. Temsirolimus and Everolimus are so-called Rapalogs, synthetic analogs of rapamycin. They bind with a high affinity to the FK506 binding protein-12 (FKBP-12) in a drug complex that directly inhibits mTOR, in the mTORC1 complex, while mTORC2 remains unaffected [122]. AZD8055 and INK128 inhibit mTOR activity as ATP-competitors, which impede mTOR in mTORC1 as well as mTORC2 [123,124].

## 8. Conclusions

HCC, the third leading cause of cancer death worldwide, often develops from primary liver lesions induced by acute liver injury, hepatitis induced by viruses, alcohol-induced as well as non-alcoholic fatty liver disease, autoimmune hepatits and various supporting factors or triggers such as drug abuse, metabolic processes or epigenetics. HCV, with the hijacking of the translational machinery for the IRES-mediated translation of its RNA genome, is an example of the many different ways that the protein expression process can be modulated under cellular stress conditions. Inflammatory processes seem to be more likely in some patients than in others, and probably manifest themselves through a combination of the above-mentioned external factors, together with internal, individual circumstances. 

According to previous reports, eIFs, particularly some eIFs, with their main regulator mTOR, seem to play a crucial role in the development of various cancer entities, such as HCC. Cell death, inflammation and compensatory proliferation provide the fundamentals. As the translation machinery, specifically, the eIFs, are modulated by multiple inducers, such as interferons, cellular stress and viral infection, it is assumed that eIFs could serve as potential future therapeutic targets as they expose attack surfaces for drugs and small molecules.

## Data Availability

No new data were created or analyzed in this study. Data sharing is not applicable to this article.

## Supplementary Materials

The following supporting information can be downloaded at: https://www.mdpi.com/article/10.3390/cells11030533/s1, Table S1: Active and beginning clinical trials against Hepatocellular Carcinoma/Cancer with the drugs Rapamycin, Temsirolimus, Everolimus, AZD8055 and INK128, mainly targeting components of the mTOR pathway; Table S2: Completed clinical trials against Hepatocellular Carcinoma/Cancer with the drugs Rapamycin, Temsirolimus, Everolimus, AZD8055 and INK128, mainly targeting components of the mTOR pathway.
