# Supplementary material for: The Translational Bridge between Inflammation and Hepatocarcinogenesis
Source: Cells. 2022 Feb 3;11(3):533. doi: 10.3390/cells11030533 (PMC8834218; doi:10.3390/cells11030533)
Supplement: Supplementary file 1 [file cells-11-00533-s001.zip › cells-1544373-supplementary.pdf]

cells MDPI

Supplemetary Table S1. Active and beginning clinical trials against Hepatocellular Carcinoma/Cancer with the drugs Rapamycin, Temsirolimus, Everolimus, AZD8055 and INK128, mainly targeting components of the mTOR pathway. Data provided by clinicaltrials.gov [121].
Abbreviations Status: R-recruiting, N -not yet recruiting, A -active

Title	Status	Conditions	Interventions	Countries
Trametinib Combined With Everolimus and Lenvatinib for Recurrent/Refractory Advanced Solid Tumors	R	HCC	Everolimus inter alia	China
Sirolimus-based Immunosuppression Treatment Regimen for Liver Transplantation	N	Liver transplantation	Sirolimus (Rapamycin), inter alia	China
MLN0128 Compared to Sorafenib in Advanced or Metastatic Hepatocellular Carcinoma	A	HCC	MLN0128, (RP2D) inter alia	USA
Targeted Therapy Directed by Genetic Testing in Treating Patients With Advanced Refractory Solid Tumors, Lymphomas, or Multiple Myeloma (The MATCH Screening Trial)	R	Liver and Intrahepatic Bile Duct Carcinoma, inter alia	Capivasertib, Copanlisib, Copanlisib Hydrochloride, Ipatasertib, PI3K-beta Inhibitor GSK2636771, Sapanisertib, Sunitinib Malate, Taselisib inter alia	USA
Risk-Based Therapy in Treating Younger Patients With Newly Diagnosed Liver Cancer	A	Hepatoblasoma	Temsirolimus, inter alia	USA, Australia, Canada, Japan

Supplementary Table S2. Completed clinical trials against Hepatocellular Carcinoma/Cancer with the drugs Rapamycin, Temsirolimus, Everolimus, AZD8055 and INK128, mainly targeting components of the mTOR pathway. Data provided by clinicaltrials.gov [121].

Title	Conditions	Interventions	Countries
Temsirolimus and Sorafenib in Advanced Hepatocellular Carcinoma	Hepatocellular Carcinoma	Temsirolimus inter alia	USA
Phase II Combination of Temsirolimus and Sorafenib in Advanced Hepatocellular Carcinoma	Hepatocellular Carcinoma	Temsirolimus inter alia	USA
Immunosuppression in Patients Undergoing Liver Transplantation for Hepatocellular Carcinoma	Hepatocellular Carcinoma	Sirolimus (Rapamycin) inter alia	Germany
Hepatocellular Carcinoma (HCC)_Torisel_	Inoperable HCC	Torisel	China
Efficacy of Sirolimus In Liver Transplantation for Hepatocellular Carcinoma (HCC)	Hepatocellular Carcinoma	Sirolimus (Rapamycin)	South Korea
Adjuvant Anti-Recurrence Treatment With Rapamycin on Early-stage HCC	Hepatocellular Carcinoma, Rapamycin	Rapamycin	China
Temsirolimus and Bevacizumab in Treating Patients With Advanced Endometrial, Ovarian, Liver, Carcinoid, or Islet Cell Cancer	Adult Hepatocellular Carcinoma, Advanced Adult Hepatocellular Carcinoma, Localized Non-Resectable Adult Liver Carcinoma, Recurrent Adult Liver Carcinoma, inter alia	Temsirolimus	USA, Canada
De Novo Sirolimus-based Immunosuppression After Liver Transplantation for Hepatocellular Carcinoma	Liver Carcinoma	Sirolimus (Rapamycin)	Canada
Sirolimus and Bevacizumab in Treating Patients With Liver Cancer That Cannot Be Removed by Surgery	Liver Cancer	Rapamycin inter alia	Singapore
Nivolumab (Opdivo) Plus ABI-009 (Nab-rapamycin) for Advanced Sarcoma and Certain Cancers	Hepatocellular Carcinoma, MTOR Activating Mutation, inter alia	Rapamycin inter alia	United States
Vaccine Therapy With or Without Sirolimus in Treating Patients With NY-ESO-1 Expressing Solid Tumors	Recurrent Hepatocellular Carcinoma, Resectable Hepatocellular Carcinoma, Sarcoma, inter alia	Sirolimus (Rapamyin) inter alia	USA

Study to Assess Safety, Pharmacokinetics, and Efficacy of Oral CC-223 for Patients With Advanced Solid Tumors, Non-Hodgkin Lymphoma or Multiple Myeloma	Multiforme, Hepatocellular Carcinoma, inter alia	CC-223	USA, France, Spain, UK
Safety, Tolerability, Pharmacokinetics (PK) and Preliminary Efficacy of Tor Kinase Inhibitor in Liver Cancer Patients	Cancer, Advanced Hepatocellular Carcinoma	AZD8055	South Korea
Lenalidomide in Combination With Bevacizumab, Sorafenib, Temsirolimus, or 5-Fluorouracil, Leucovorin, Oxaliplatin (FOLFOX)	Advanced Cancers	Temsirolimus inter alia	USA
Everolimus and Pasireotide (SOM230) in Patients With Advanced or Metastatic Hepatocellular Carcinoma	Advanced Adult Hepatocellular Carcinoma	Everolimus inter alia	USA
Antiangiogenic Treatment of Hepatocellular Cancer With Bevacizumab and RAD001	Hepatocellular Carcinoma	Everolimus	Germany
RAD001 in Advanced Hepatocellular Carcinoma	Hepatocellular Carcinoma	RAD001 (Everolimus)	USA
Study of (1) Everolimus, (2) Estrogen Deprivation Therapy (EDT) With Leuprolide + Letrozole and (3) Everolimus + EDT in Patients With Unresectable Fibrolamellar Hepatocellular Carcinoma (FLL-HCC)	Fibrolamellar Carcinoma, Fibrolamellar Liver Cancer	Everolimus, combination of everolimus inter alia	USA
Global Study Looking at the Combination of RAD001 Plus Best Supportive Care (BSC) and Placebo Plus BSC to Treat Patients With Advanced Hepatocellular Carcinoma.	Carcinoma	Everolimus, Everolimus Placebo	USA, Australia, Austria, Belgium, Canada, China, France, Germany, Greece, Hungary, Israel, Italy, Japan, South Korea, Spain, Thailand
Sorafenib Tosylate With or Without Everolimus in Treating Patients With Localized, Unresectable, or Metastatic Liver Cancer	Liver Cancer	Everolimus, inter alia	Austria, Hungary, Switzerland
Circulating Tumor Cells and Tumor DNA in HCC and NET	Carcinoma, Hepatocellular, Neuroendocrine Tumors	Everolimus inter alia	Denmark

Rapamycin With Grapefruit Juice for Advanced Malignancies	Tumors, Neoplasm Metastasis	Rapamycin (Sirolimus)	USA
A Study of CD45RA+ Depleted Haploidentical Stem Cell Transplantation in Children With Relapsed or Refractory Solid Tumors and Lymphomas	Hepatic Tumor, inter alia	Sirolimus (Rapamycin) inter alia	USA
Pasireotide, Everolimus and Selective Internal Radioembolization Therapy for Unresectable Hepatic Metastases	Neuroendocrine Tumors	Everolimus inter alia	USA

121. Zarin, D.A.; Tse, T.; Williams, R.J.; Califf, R.M.; Ide, N.C. The ClinicalTrials.Gov Results Database — Update and Key Issues. *N Engl J Med* **2011**, *364*, 852–860, doi:10.1056/NEJMsa1012065. (accessed 25.01.2022)
